# Gantry angle determination during arc IMRT: evaluation of a simple EPID‐based technique and two commercial inclinometers

**DOI:** 10.1120/jacmp.v13i6.3981

**Published:** 2012-11-08

**Authors:** Pejman Rowshanfarzad, Mahsheed Sabet, Daryl J. O'Connor, Peter M. McCowan, Boyd M.C. McCurdy, Peter B. Greer

**Affiliations:** ^1^ School of Mathematical and Physical Sciences University of Newcastle Newcastle NSW Australia; ^2^ Department of Radiation Oncology Calvary Mater Newcastle Hospital, Newcastle School of Mathematical and Physical Sciences University of Newcastle Newcastle NSW Australia; ^3^ Department of Physics and Astronomy University of Manitoba, Winnipeg Division of Medical Physics CancerCare Manitoba Winnipeg MB Canada; ^4^ Department of Radiology University of Manitoba Winnipeg MB Canada

**Keywords:** gantry angle, arc IMRT, quality assurance, inclinometer

## Abstract

The increasing popularity of intensity‐modulated arc therapy (IMAT) treatments requires specifically designed linac quality assurance (QA) programs. Gantry angle is one of the parameters that has a major effect on the outcome of IMAT treatments since dose reconstruction for patient‐specific QA relies on the gantry angle; therefore, it is essential to ensure its accuracy for correct delivery of the prescribed dose. In this study, a simple measurement method and algorithm are presented for QA of gantry angle measurements based on integrated EPID images acquired at distinct gantry angles and cine EPID images during an entire 360° arc. A comprehensive study was carried out to evaluate this method, as well as to evaluate two commercially available inclinometers (NG360 and IBA GAS supplied in conjunction with popular array dosimeters ${\text{Delta}}^{4}$ and ${\text{MatriXX}}^{\text{Evolution}}$, respectively) by comparing their simultaneous angle measurement results with the linac potentiometer readouts at five gantry speeds. In all tested measurement systems, the average differences with the reference angle data were less than 0.3° in static mode. In arc mode, at all tested gantry speeds the average difference was less than 0.1° for the IBA GAS and the proposed EPID‐based method, and 0.6° for the NG360 after correction for the inherent systematic time delay of the inclinometer. The gantry rotation speed measured by the three independent systems had an average deviation of about 0.01°/s from the nominal gantry speed.

PACS numbers: 06.30.Bp; 87.56.Fc; 87.56.‐v

## I. INTRODUCTION

Intensity‐modulated arc therapy (IMAT) is a novel form of radiotherapy treatment that allows the radiation dose to be delivered in one or two arcs.^1^, ^2^

This technique offers precise target coverage using lower target doses and shorter delivery times compared with intensity‐modulated radiotherapy (IMRT). The method is more complex than IMRT since the gantry speed, dose rate, and the MLC‐defined field shape are varied during the delivery.^1^, ^3^, ^4^ Therefore, the QA programs developed for IMRT do not sufficiently address the requirements for IMAT.^(5)^ Due to the increasing worldwide interest in this technique, it is essential to develop more sophisticated QA programs that take all components that affect the accuracy of IMAT delivery into account.^(2)^

One of the major considerations for implementation of new treatment techniques is verification of the predicted doses. In the case of IMAT treatments, this involves gantry angle‐resolved dosimetric information.^5^, ^8^ Misalignment of the linac angular settings could severely affect the dose distribution of an IMAT plan delivery and have serious clinical consequences due to the steep dose gradients and complex MLC shapes.^(9)^

In routine QA of linacs, a level is positioned on a flat surface of the gantry head close to the graticule and the gantry is rotated until the bubble settles at the center. Using this method, the gantry angle indicator can be checked only for cardinal angles, and the flatness of the surface usually remains unchecked.^9^, ^10^ Another method suggested for QA of the angle indicator is to perform a star shot on film at distinct static gantry angles and determine the angle based on the film setup.^(9)^ This method is not suitable for testing in arc mode, and introduces the difficulties of film measurements and processing. A ±1° limit has been recommended as the action level for the gantry angle readout system by the AAPM Task Group 40.^(11)^

In a study on linac gantry angles during arc treatments, cine images were acquired during IMAT deliveries using an electronic portal imaging device (EPID) with a specially designed phantom in the beam. The phantom consisted of a pair of wires wound around a cylinder. The accuracy of gantry angles recorded in the header of these DICOM images was investigated by comparison to the angles derived by following the points of intersection of wires in each image.^12^, ^13^ It was found that the same angle may be repeated in headers of successive images due to the low frequency of angle readout update.

Other proposed methods for the determination of gantry angle were based on EPID images of phantoms containing a number of radio‐opaque fiducial markers. Edge detection filters, or thresholding methods, were used to detect the marker edges, and numerical optimization functions were applied to find the center of each ball bearing. Gantry angles were derived from the relative positions of the markers in the images.^14^, ^15^

Due to the importance of dose verification, especially in arc treatments, in the present study three independent measurement systems have simultaneously been used for gantry angle determination. A simple and easy‐to‐use phantom is suggested, and a fast accurate algorithm is used to determine the gantry angle during a 360° arc using cine EPID images. The measurement results are evaluated by comparison to the linac log files used as reference. Furthermore, the accuracies of two types of inclinometers supplied with commercial array dosimeters, which are commonly used for pretreatment verification of IMAT plans, have been investigated.

## II. MATERIALS AND METHODS

**Figure 1:**
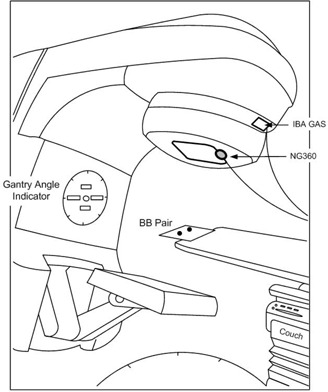
Schematic illustration of the experimental setup for three methods of simultaneous gantry angle measurements. The ball bearings are positioned in the beam at the isocenter level and two inclinometers are fixed to the gantry head.

A Varian Trilogy linear accelerator (Varian Medical Systems, Palo Alto, CA) was used for the experiments. These systems are equipped with two encoding potentiometers that replicate each other and provide signals linearly proportional to the gantry angle. The signals are sent to a digital readout system and, as a result, the gantry angle is demonstrated on the angle indicator (Fig. 1) and the console. In addition, the gantry angles detected by the potentiometers are saved in the linac delivery log files (MLC DynaLog files, referred to as DynaLog files throughout the text). These files are generated by the Varian MLC control software and updated every 0.05 s and are only accessible after the delivery is completed. In the present study, the gantry angles saved in the DynaLog files were used as reference. The accuracy of potentiometer measurements was first evaluated by moving the gantry to cardinal angles using a spirit level placed on a flat surface of the head and comparing to the potentiometer readouts.

### A. Inclinometer measurements

Two types of inclinometers have been studied in the present work. They are supplied in conjunction with well‐known commercially available array dosimeters that are commonly used for dosimetric verification of IMRT/IMAT plans.

*Nordic Transducer NG360* (Hadsund, Denmark): This digital inclinometer is supplied with the ${\text{Delta}}^{4}$ dosimetry device (ScandiDos AB, Uppsala, Sweden) which contains two orthogonal matrices of diodes enclosed in a cylindrical phantom. The NG360 is a liquid capacitive‐based inclinometer that is attached to the gantry, and has the ability of measuring the tilt angle with respect to gravity over the range of 360°. The inclinometer has 0.01° resolution and its maximum readout frequency is 1 Hz. The accuracy of its measurements is stated to be ± 0.25°.^(16)^

The NG360 was firmly bolted to a steel frame and the frame was attached to the gantry head through an accessory tray slot such that it could not move during gantry rotation (Fig. 1). It was aligned at zero gantry angle (IEC scale) before each series of measurements by comparison to the linac gantry angle indicator. The NG360 was connected to a PC through a converter, and the collected data were processed by the accompanying program supplied by the vendor (GetAngle.exe).

***IBA Gantry Angle Sensor*** (IBA GAS; IBA Dosimetry GmBH, Schwarzenbruck, Germany): This inclinometer is supplied with the ${\text{MatriXX}}^{\text{Evolution}}$ dosimetry device (IBA Dosimetry GmBH, Germany) which includes a two‐dimensional array of ionization chambers and is used for pretreatment verification of IMRT/IMAT plans. The snapshots are recorded in movie mode with their corresponding measured angles, and are thus used for three‐dimensional dose calculations, as well as corrections for optimization of the angular dependency of the array. The accuracy of its measurements is stated to be ± 0.6°.^(17)^

The IBA GAS was attached to the gantry head (Fig. 1) and leveled by two locking screws. It was adjusted to the linac gantry angle indicator at 0° and 90° angles, and was finally aligned using the four setup LEDs on the device. The sampling frequency was set to 1 Hz.

The MatriXX was not positioned in the beam, but was connected to the GAS, power source, and a PC. Measurement results were processed using OmniPro‐I'mRT v.1.7.0007 software (IBA Dosimetry GmbH, Germany) which was installed on the PC.

It must be noted that the data measured by both inclinometers were only accessible after the delivery was completed.

### B. EPID‐based angle measurement setup

Another independent measurement method used in this study was based on EPID images of a dual ball bearing (BB) phantom. Two 4.8 mm diameter tungsten carbide BBs were fixed on a thin plastic plate 14 cm apart and positioned in the beam at the isocenter level. An amorphous silicon aS1000 EPID attached to the linac was used for image acquisition. The phantom was fixed to the top edge of the couch (toward the gantry) so that the shadow of the couch could not affect the BB images (Fig. 1). Irradiations were carried out using 6 MV treatment beams at a variety of doses and delivery rates to perform the test at different gantry speeds. The EPID has an array of 1024×768 pixels in a $40\times 30{\;\text{cm}}^{2}$ area and produces images in DICOM format. The imager was remotely retracted at zero gantry angle using the imager control box in the treatment console before each series of acquisitions to ensure that the detector was in a reproducible location.

The centers of the BBs were automatically detected in each image using an algorithm already developed and explained in detail by Rowshanfarzad et al.^18^, ^19^, ^20^ in the MATLAB programming language (The MathWorks Inc., Natick, MA) with minor modifications. The algorithm finds the position of BBs by determining the minimum signal value in a region of interest with subpixel accuracy. Variation of the distance between the BBs (d) in cine images indicated the changes in gantry angle. The gantry angle for each projection image was determined using a calibration curve already derived from images acquired at distinct gantry angles (from ‐180° to 180° in 30° intervals), which yielded the gantry angle as a function of distance (d).

### C. Measurement methods

The experiments were performed in static and arc modes. The three independent systems were simultaneously used for gantry angle measurements.

#### C.1 Measurements in static mode

The readouts of inclinometers and EPID‐based angle measurements were first evaluated by comparison of the results for distinct static gantry angles with the linac angle indicator (which originates from the potentiometer). Measurements were made at gantry angles from ‐180° to 180° in 30° intervals, and each series was tested three times to ensure the reproducibility. EPID images were acquired in integrated mode using 100 MU irradiations at 300 MU/min delivery rate.

#### C.2 Measurements during arc delivery

In order to make measurements that cover the whole range of possible gantry speeds (1.5°, 2°, 3°, 4°, and 5° per second), various MUs were delivered at a nominal rate of 300 MU/min (Table 1) during 360° arcs, while cine EPID images were acquired in service mode at an acquisition rate of 7.5 frames per second (5 frames per image). The imager and both inclinometers were started simultaneously ~6 s before the beam was turned on and continued to measure a few seconds after the beam was turned off. The angle data in the linac DynaLog files were used as reference for comparisons.

**Table 1:** Measurement settings for beam delivery at various gantry speeds during 360° arcs.

*Gantry Speed (degrees/s)*	*5*	*4*	*3*	*2*	*1.5*
MU Setting	360	450	600	900	1200
Beam‐on time (s)	72	90	120	180	240

**Figure 2:**
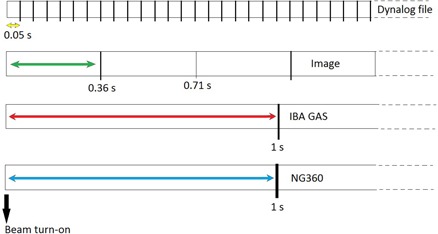
Schematic diagram showing the timing for data acquisition with the measurement devices used in this study.

With the start of beam delivery, the DynaLog file begins to record the angles (with 20 Hz frequency) and the EPID starts to acquire images. To synchronize the BB phantom method with the DynaLog file, half of the time required for the acquisition of the first image (~ 0.36 s) was considered as the start of EPID imaging and its angle was compared with the corresponding data point in the DynaLog file. The timing for data acquisition is shown in a diagram in Fig. 2. Furthermore, as the beam is turned on, the inclinometer readouts start to change with gantry rotation. The angles measured by the inclinometers were compared with those recorded in the DynaLog file at corresponding times.

The gantry speeds measured by the three methods were derived from the datasets $\left(\dot{\theta }=\frac{\Delta \theta }{\Delta t}\right)$ and compared with the nominal gantry speeds.

## III. RESULTS

### A. calibration curve for the EPID‐based method

**Figure 3:**
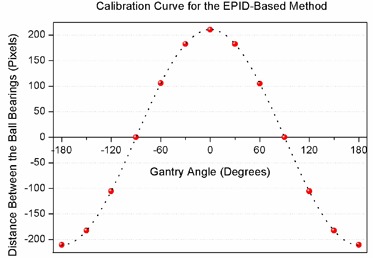
Results of the algorithm for angle measurements at distinct gantry angles. The dotted line shows the curve fitted through the data points that was used to derive the calibration function for the EPID‐based measurement method.

The algorithm results for the distance between the BB pair at distinct gantry angles are shown in Fig. 3. The negative values indicate the change in the relative positions of the BBs in image projections during gantry rotation.

A curve was fitted through the data points and was used to determine the calibration function to find the angle corresponding to each BB distance for the entire 360° arc (Eq. (1)):

(1) $$\theta_{d}=\frac{1}{0.01746}\times (\sin^{-1}(\frac{d}{210.7})-1.571)$$

where $\theta_{d}$ is the gantry angle in degrees and *d* is the pixel distance between the BBs.

### B. Static gantry measurements

The evaluation of linac potentiometer accuracy showed an average 0.02° difference between the angle settings using the spirit level and the potentiometer‐based angle indicator for cardinal angles.

**Figure 4:**
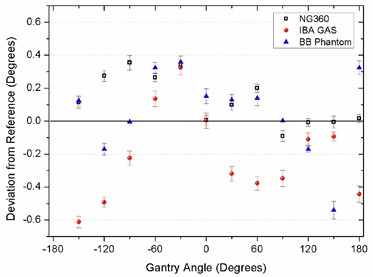
Comparison of the three independent measurement systems with the reference gantry angle indicator for distinct static angles.

The angles measured by both inclinometers and the EPID‐based method at distinct angles were compared with the reference gantry angle indicator values which are read out from the linac potentiometer (Fig 4).

The average differences (± 1 SD) for the NG360, IBA GAS, and the BB phantom measurements with the reference were 0.15° ± 0.13°, ‐0.29° ± 0.18°, and 0.20° ± 0.16°, respectively.

### C. Measurements during arc

**Figure 5:**
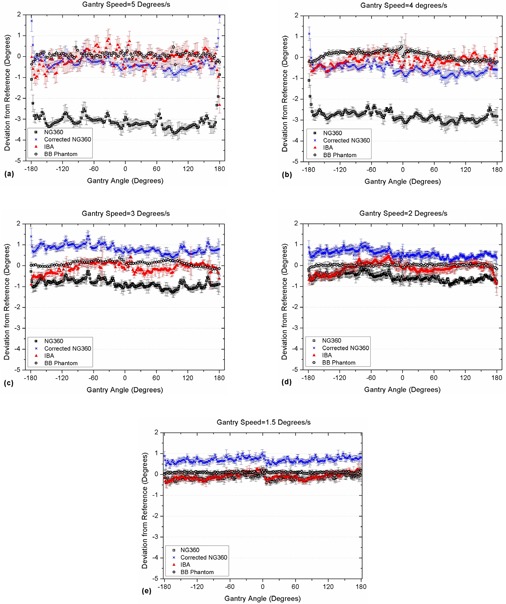
Deviations of the results of different angle measurement methods from the linac DynaLog files over 360° arcs in: (a) 5°, (b) 4°, (c) 3°, (d) 2°, and (e) 1.5°/s nominal gantry rotation speeds.

Comparison of the experimental results during beam delivery in 360° arcs with the linac DynaLog files (used as reference) revealed some deviations in all measurements. The results for NG360 were discussed with the manufacturer and an inherent time delay of 0.56 s was reported by the company. Detailed results for each measurement system (including the corrected NG360 readings) are shown in Fig. 5 for each gantry speed and the average deviation for each speed (± 1 SD) is given in Table 2.

**Table 2:** Average deviations of the measured gantry angles over 360° arcs rotating at different speeds compared with the linac DynaLog files, and the gantry speeds measured using the three independent measurement methods. All values are given ± 1 SD.

*Nominal Gantry Speed (Degrees/s)*	*Average Deviation from Reference (Degrees)*				*Measured Average Speed (Degrees/s)*		
	*NG360*	*NG360 (corrected)*	*IBA*	*BB Phantom*	*NG360*	*IBA*	*BB Phantom*
5.0	‐3.11±0.37	‐0.31±0.37	‐0.12±0.41	0.06±0.14	5.00±0.22	4.99±0.14	4.98±0.21
4.0	‐2.81±0.25	‐0.57±0.25	‐0.14±0.24	0.10±0.20	3.98±0.15	3.99±0.17	3.98±0.16
3.0	‐0.87±0.19	0.81±0.19	‐0.11±0.23	0.10±0.12	3.00±0.14	3.00±0.10	3.00±0.12
2.0	‐0.56±0.16	0.55±0.16	‐0.12±0.24	0.01±0.05	2.00±0.08	1.99±0.08	1.99±0.14
1.5	‐0.14±0.11	0.70±0.11	‐0.08±0.11	0.09±0.03	1.49±0.06	1.50±0.09	1.49±0.07

**Figure 6:**
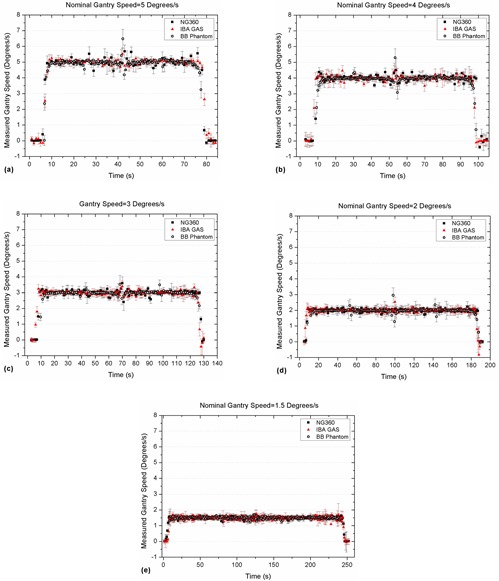
Measured gantry rotation speeds from the angle datasets during arc using the three simultaneous measurements for: (a) 5°, (b) 4°, (c) 3°, (d) 2°, and (e) 1.5°/s nominal speeds.

Gantry speeds were also determined from the angle measurement data during arcs. The results are compared in Fig. 6 for different nominal gantry rotation speeds. Details are given in Table 2.

## IV. DISCUSSION

Implementation of new radiotherapy techniques requires accurate and efficient quality assurance procedures. Characterization of the changes in gantry angle is essential for the QA of machine performance in IMAT deliveries and also for real‐time dosimetry or three‐dimensional dose reconstruction.

In this study, an EPID‐based measurement method with a simple phantom consisting of two ball bearings was proposed in conjunction with a robust algorithm to measure the gantry angle during an entire 360° arc with subpixel accuracy. To achieve a highly accurate algorithm and more reliable results, it is recommended to position the BBs as far apart as possible and use most of the active length of the detector. The ball bearings should be large enough to involve sufficient number of pixels, and should have high density to provide the required level of image contrast for data processing.

Results of this method were compared with two existing commercial devices currently used for dosimetry, and the simultaneous measurements made by the three independent systems were evaluated by comparison to the reference data provided by the linac potentiometer. Using the DICOM image headers as reference could be an option, but was abandoned since up to three images in a dataset had the same angle in their headers. This confirmed previous findings by Ansbacher et al.^(12)^

Results of angle measurements in static mode showed that the NG360 inclinometer provided the closest data to the linac potentiometer, while in arc mode it had the largest deviations from the potentiometer at all gantry speeds. This was attributed to the changes in the capacity of the liquid‐based sensor system during gantry rotation, in addition to a possible delay in the readout communications. After correction for the 0.56 s systematic time delay as recommended by the manufacturer, the deviation of readings with reference data became less than 1° for all gantry speeds, although the correction was more effective for higher gantry speeds (4° and 5°/s).

According to Fig. 5, although the average IBA GAS inclinometer readings are in good agreement with the reference data, there are fluctuations in their readouts at high gantry speeds. However, the range of variations is generally within the tolerance limit for gantry angle (±1°). The reason for such noisy results is not clear due to the lack of information about the measurement mechanism and the sensor structure.

Comparison of the gantry rotation speed measured by the three independent methods showed that, on average, all systems had a deviation of up to 0.01°/s from the nominal gantry speed. A jump was observed around the middle of beam delivery in Fig. 6 (~ zero gantry angle), which was attributed to the mechanical structure of Varian linacs. A similar effect has been reported in the literature.^19^, ^21^, ^22^

The angle measurements with the EPID‐based BB phantom method and algorithm used in this work were within 0.1° of the reference data at all gantry speeds. The measured data were not noisy and required no time delay corrections. More importantly, the angle information is derived separately from the analysis for each image, and there is no need for synchronization of the gantry angles and images, which makes this method superior to the indirect readouts from the potentiometer or inclinometers. However, setting up the BB phantom for pretreatment dose verification or real‐time dosimetry without modification of the MLC and jaw positions is not feasible. One suggested method would be to open a pair of the outer leaves (far from the treatment field) and set the ball bearings at the isocenter level such that their EPID images could be acquired to provide the angle data for each image independently. The dose corresponding to this part of the image could easily be excluded for dose reconstruction. However, using the inclinometers (for real‐time investigations) or the linac DynaLog files for pretreatment dose verification or real‐time dosimetry may be more appropriate options.

## V. CONCLUSIONS

The present study showed that the proposed EPID‐based BB phantom and algorithm can accurately measure the gantry angles for static and arc deliveries and be used as a reliable method for gantry angle measurements. At high gantry speeds, the IBA GAS inclinometer provides noisy readings within the gantry angle tolerance limits and the NG360 inclinometer data fit within the ±1° tolerance levels after a time delay correction.

## ACKNOWLEDGMENTS

This work was supported by the National Health and Medical Research Council Grant (Grant No. 569211). The authors would like to thank Dennis Pomare, Christopher Lewis, and Karl Stansfield from Calvary Mater Newcastle Hospital for their help and support, and Thomas Matzen from Scandidos for his cooperation in the investigation of NG360. The first author gratefully acknowledges the award of the UNIPRS scholarship from the University of Newcastle, Australia.
